# Investigating the Antifungal Mechanism of Action of Polygodial by Phenotypic Screening in *Saccharomyces cerevisiae*

**DOI:** 10.3390/ijms22115756

**Published:** 2021-05-28

**Authors:** Purity N. Kipanga, Liesbeth Demuyser, Johannes Vrijdag, Elja Eskes, Petra D’hooge, Josphat Matasyoh, Geert Callewaert, Joris Winderickx, Patrick Van Dijck, Walter Luyten

**Affiliations:** 1 Faculty of Pharmaceutical Sciences, KU Leuven, 3000 Leuven, Belgium; 2 Department of Biology, Animal Physiology and Neurobiology, KU Leuven, 3000 Leuven, Belgium; walter.luyten@kuleuven.be; 3 Laboratory of Molecular Cell Biology, Department of Biology, KU Leuven, 3000 Leuven, Belgium; liesbeth.demuyser@kuleuven.be (L.D.); elja.eskes@kuleuven.be (E.E.); patrick.vandijck@kuleuven.be (P.V.D.); 4 VIB-KU Leuven Center for Microbiology, 3000 Leuven, Belgium; 5 Department of Chemistry, Molecular Design and Synthesis, KU Leuven, 3000 Leuven, Belgium; Johannes.Vrijdag@martens.be; 6 Department of Biology, Functional Biology, KU Leuven, 3000 Leuven, Belgium; joris.winderickx@kuleuven.be; 7 The Yeast Hub Lab, KU Leuven, Campus Kulak, 8500 Kortrijk, Belgium; petra.dhooge@kuleuven.be (P.D.); geert.callewaert@kuleuven.be (G.C.); 8 Department of Chemistry, Egerton University, Njoro 20107, Kenya; jmatasyoh@egerton.ac.ke

**Keywords:** Polygodial, antifungal, *Saccharomyces cerevisiae*, haploid deletion mutant library, V-ATPase, vacuolar pH, ubiquitin, TORC1 signaling, Ca^2+^ signaling and Ca^2+^ homeostasis

## Abstract

Polygodial is a “hot” peppery-tasting sesquiterpenoid that was first described for its anti-feedant activity against African armyworms. Using the haploid deletion mutant library of *Saccharomyces cerevisiae*, a genome-wide mutant screen was performed to shed more light on polygodial’s antifungal mechanism of action. We identified 66 deletion strains that were hypersensitive and 47 that were highly resistant to polygodial treatment. Among the hypersensitive strains, an enrichment was found for genes required for vacuolar acidification, amino acid biosynthesis, nucleosome mobilization, the transcription mediator complex, autophagy and vesicular trafficking, while the resistant strains were enriched for genes encoding cytoskeleton-binding proteins, ribosomal proteins, mitochondrial matrix proteins, components of the heme activator protein (HAP) complex, and known regulators of the target of rapamycin complex 1 (TORC1) signaling. WE confirm that polygodial triggers a dose-dependent vacuolar alkalinization and that it increases Ca^2+^ influx and inhibits glucose-induced Ca^2+^ signaling. Moreover, we provide evidence suggesting that TORC1 signaling and its protective agent ubiquitin play a central role in polygodial resistance, suggesting that they can be targeted by polygodial either directly or via altered Ca^2+^ homeostasis.

## 1. Introduction

**Figure 1:**
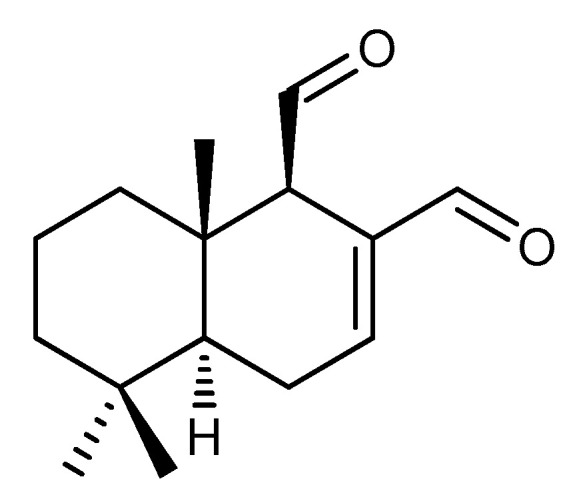
The chemical structure of polygodial.

Apart from the anti-feedant activity against a number of pests [1,2,3], polygodial (Figure 1) has been reported to possess biological activities such as antifungal [4,5,6], antibacterial [7], anti-tumor [8], larvicidal [9], antihelminthic [10], antifouling [11], anti-inflammatory [12], analgesic [13], antitrypanosomal, and antileishmanial activities [14]. As a bioactive constituent, it is produced in several plants of the Canellaceae [1], Polygonaceae [15], and Winteraceae [16] families, as well as in marine animals such as sponges and molluscs [17,18]. Recently, we isolated polygodial from *Warburgia ugandensis* Sprague subspecies *ugandensis* (Canellaceae) and showed its activity against *Candida albicans* [6]. Specifically, we showed that low concentrations of polygodial are not only active against the planktonic forms of *C. albicans*, but that the compound is also active against its biofilm forms (biofilm inhibiting concentration: BIC_50_ =10.8 ± 5 μg/mL); which are a major menace to public health due to their increased resistance to currently available antifungals [19]. These interesting findings prompted us to elucidate the antifungal mechanism of action (MOA) of polygodial. 

Earlier antifungal MOA studies have led to different conclusions. Taniguchi and colleagues showed that polygodial disrupted the cell membrane in *S. cerevisiae*, resulting in loss of cellular content [20]. Although these authors also showed that polygodial inhibited both respiration and the synthesis of cellular macromolecules such as DNA, RNA, proteins, and polysaccharides, they concluded that these were likely secondary effects of the cell damage caused by polygodial since the inhibition of these macromolecules was not specific. In addition, they also ruled out the mitochondria as the primary target since only exogenous respiration and not endogenous respiration was affected. Later, Lunde and Kubo intimated a more complex mechanism of polygodial’s activity [21]. Specifically, they showed, using isolated membrane fractions of *S. cerevisiae*, that polygodial rapidly inhibited mitochondrial ATPase at similar concentrations (3.5 µg/mL) as its minimum fungicidal concentration. Polygodial also showed a low inhibitory activity against the vacuolar (V-)ATPase. Further, they observed that while cytoplasmic petite mutants that lack mitochondrial DNA (*rho^-^*) and respiratory-deficient petite mutants (*rho*^0^) were resistant to polygodial at 6.25 µg/mL, the wild type (WT) was sensitive to this concentration. They thus concluded that polygodial’s major target was the mitochondrial ATPase, whose inhibition results in low cellular ATP production, explaining polygodial’s fungicidal activity. Castelli’s group [22] argued that a more significant effect of polygodial was the uncoupling of ATP synthesis from the electron transport and not the direct inhibition of the mitochondrial ATPase. Other authors have suggested targets of polygodial such as the plasma membrane H^+^-ATPase [23], cellular glutathione [24], and the vacuolar membrane [25].

We explored the antifungal MOA of polygodial on *C. albicans* using a genome-wide growth-based screening of the haploid deletion strain library of *S. cerevisiae* [26,27]. *S. cerevisiae* was selected as a model organism for a number of reasons, some of which are its similarities to *C. albicans* (e.g. many features of pathogenic fungi such as biofilm formation, maturation, and drug resistance are conserved in *S. cerevisiae*) and the availability of complete genomic libraries; which are absent for *C. albicans*, that facilitate genome-wide screening studies [28,29]. Such a screen of a strain library in the presence of a bioactive compound can provide important functional information based on the scoring of these strains for hypersensitivity and resistance to antifungal compounds. We show that the vacuolar ATPase is involved in buffering the cell against a polygodial insult and discuss the interesting link between the cytoskeletal proteins and the V-ATPases. Finally, we propose that the target for polygodial may act on TORC1 signaling, either directly or via disturbance of the Ca^2+^ homeostasis.

## 2. Results

### 2.1. Phenotypic Screening of Mutants Treated with Polygodial

**Figure 2:**
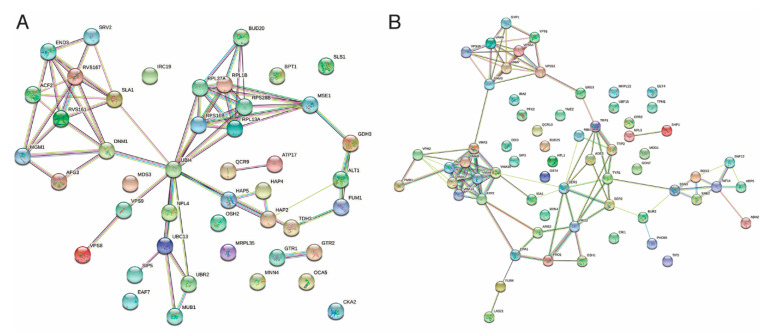
A clustering visualization of the 47 polygodial hyper-resistant (**A**) and 66 polygodial hypersensitive (**B**) strains using the STRING database. The colored circular nodes represent proteins while the edges joining the nodes represent protein–protein interactions, either established or predicted interactions.

We tested the effect of polygodial on the growth of 4828 deletion mutants in the *S. cerevisiae* haploid MATα (BY4742) deletion library. Two concentrations of polygodial (2 and 6 µg/mL) were used to select the strains that were either sensitive or resistant to polygodial by comparing their growth with that of the wild type (WT) strain *S. cerevisiae* BY4742 and ranking this growth as described in Section 4.1. From this phenotypic screening, we identified 233 strains displaying polygodial resistance and 135 strains that were polygodial-sensitive. Of these, 47 strains were classified as hyper-resistant (ranked as 6) and 66 strains as hypersensitive (ranked as 0 or 1) to polygodial. A representation of how the screening was performed and how growth was used as a read-out to enable ranking is shown in the supplemental Figure S1. We used the Saccharomyces Genome Database (SGD) [30] gene ontology (GO) Term Finder and the functional specification (FunSpec) software [31] as well as manually retrieving information of annotated genes from SGD to classify the strains into groups based on the functional roles, biochemical properties, and localization of their deleted genes (Tables S1 and S2). Next, we used the search tool for the retrieval of interacting genes/proteins (STRING) [32] database to determine the connections between the different encoded gene functions based on physical and genetic interactions, predicted interactions, co-expression, or protein homology. A visualization of the network obtained for the 47 hyper-resistant and 66 hypersensitive gene functions is shown in Figure 2. The networks derived from all retrieved sensitive or resistant gene functions is given in Figures S2 and S3. 

The cluster and gene enrichment analysis for the hyper-resistant genes (Figure 2A) pointed towards cytoskeleton reorganization and endocytosis, the assembly of ribosome subunits and translation, respiratory gene expression mediated by the HAP complex, and protein ubiquitination and degradation as important processes. In fact, the cluster analysis indicated the latter to play a central role given that the gene UBI4, which encodes ubiquitin, defines a central node that connects to all of the other processes. In addition, the enrichment analysis also retrieved three components that constitute the GSE/EGO complex. This complex is localized at late endosomes and the vacuolar membrane where it serves to sense amino acids, activate TORC1 [33]; a central regulator of cell growth and signal to sort the general amino acid permease Gap1 to the plasma membrane when amino acids become limiting [34,35]. Interestingly, also Pib2 was retrieved among the polygodial resistant strains. This protein is a glutamine sensor that acts independently of the GSE/EGO complex to activate TORC1 [36]. Compelling, TORC1 has been linked to endocytosis [37], ribosome biogenesis [38], and the HAP complex [39,40] and therefore TORC1 may represent a second central node in polygodial resistance. The TORC1 node is closely linked to the UBI4 node since ubiquitin was shown to exert a positive effect on TORC1 by preventing degradation of the TORC1 subunit Kog1 under stress conditions [41,42]. Thus, since both deletion of UBI4 and abrogation of TORC1 signaling trigger polygodial hyper-resistance, the data of this analysis suggest that both could be the actual targets of polygodial. 

For the hypersensitive gene functions (Figure 2B), four main clusters can be seen in the network. These are formed by components of the vacuolar V-ATPase, components required for amino acid biosynthesis, components involved in nucleosome mobilization or the formation of the mediator complex that is required for transcription initiation, as well as components involved in endosome trafficking. Not surprisingly, these components are also specifically enriched when analyzing the pool of polygodial sensitive genes using the FunSpec software as shown in Table 1. The data suggest that cells want to enhance these processes to counteract the effect of polygodial treatment, but that failure to do so results in polygodial sensitivity.

### 2.2. Vacuolar pH Measurements in the Presence of Polygodial

**Figure 3:**
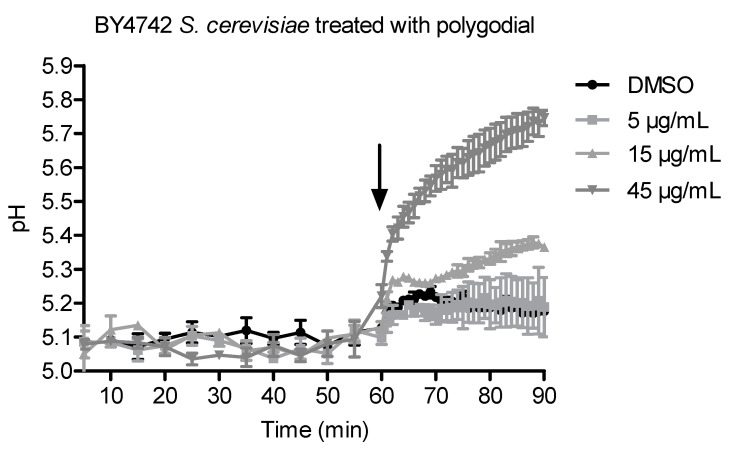
Vacuolar pH measurements in *S. cerevisiae* WT in the presence of the vehicle (DMSO) or 5, 15, and 45 µg/mL polygodial, mean with standard error of the mean (SEM), *n* = 2. Baseline measurements were first taken for 1 h in the presence of calibration buffers, then polygodial and DMSO were added in their respective wells, and the plate was further measured for 30 min. The arrow indicates addition of polygodial shortly after 60 min.

The vacuolar membrane ATPase (*VMA*) genes were highly enriched in the hypersensitive mutants (Table 1). Since the vacuolar ATPase is involved in vacuolar acidification, we examined whether and how polygodial affects vacuolar pH by measuring vacuolar pH in polygodial-treated WT *S. cerevisiae* (Figure 3). We observed that vacuolar pH values increased dose-dependently with higher concentrations of polygodial, implying that polygodial increases vacuolar pH. Continuous exposure to high concentrations of polygodial can thus disrupt the normal functioning of the vacuole.

### 2.3. Effects of Polygodial on Yeast Ca^2+^ Homeostasis

It is well established that pH homeostasis, vacuolar functioning and Ca^2+^ signaling are closely linked in yeast [43,44]. Because extracellular Ca^2+^ may exert a protective effect against the membrane-damaging action of polygodial [45] and because Ca^2+^ was shown to act as an important second messenger for TORC1 activation [46,47], we were interested in determining the influence of polygodial on intracellular Ca^2+^ homeostasis. For this purpose, carbohydrate-starved BY4741 yeast cells were challenged by glucose re-addition in aequorin-based experiments. Glucose re-addition to starved yeast cells has been shown to produce a transient elevation in calcium concentration (TECC) response that is dependent on Ca^2+^ influx [48]. 

**Figure 4:**
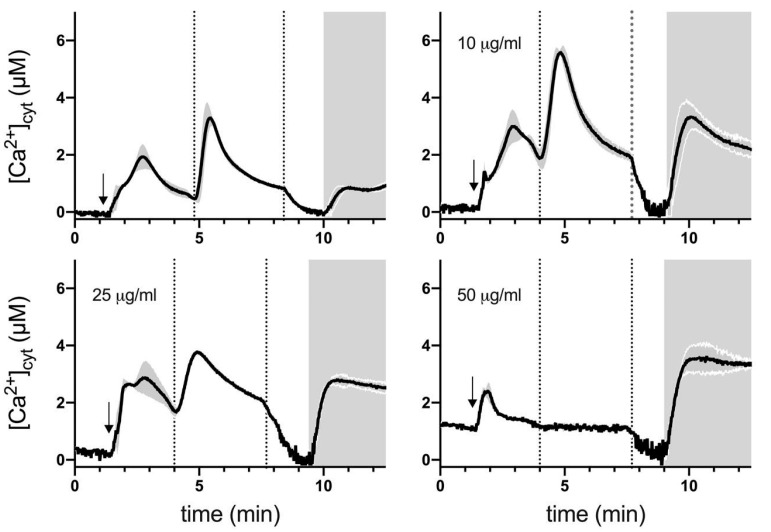
Effects of polygodial on yeast Ca^2+^ homeostasis. Averaged Ca^2+^ transient (grey shaded region either side of the Ca^2+^ transient trajectory marks SEM, *n* = 3) of wild-type BY4741 yeast cells after 30 min pre-treatment with vehicle (DMSO) (upper left panel) or 10, 25, and 50 μg/mL polygodial (as indicated). Cells were initially perfused with Ca^2+^-free starvation medium and then transferred to a 10 mM external Ca^2+^ medium (indicated by arrow) followed by re-addition of 80 mM glucose (dashed vertical lines indicate perfusion with glucose that results in a TECC response). Thereafter, cells were briefly exposed to Ca^2+^-free medium prior to membrane permeabilization using Triton X-100 (grey shaded zone correlates with membrane permeabilization).

As shown in Figure 4, in control (DMSO vehicle treated) cells, addition of 10 mM external Ca^2+^ induced a rapid small [Ca^2+^]_cyt_ increase and glucose re-addition (80 mM) generated a characteristic TECC response as observed previously [49]. Membrane permeabilization after the TECC response induced a small Ca^2+^ transient, reflecting the release of Ca^2+^ mainly sequestered in the vacuole and Golgi during the TECC response [49]. A 30 min pre-treatment with 10 μg/mL polygodial had no significant effect on baseline Ca^2+^ levels but cytosolic Ca^2+^ responses following 10 mM external Ca^2+^ addition or glucose re-addition increased significantly compared to control cells. In addition, the Ca^2+^ transient following membrane permeabilization displayed about 4-fold higher peak. This finding suggests that Ca^2+^ sequestration is not compromised but that increased Ca^2+^ influx may underlie the observed Ca^2+^ changes in polygodial-treated cells. Thirty minute pre-treatment with 25 μg/mL polygodial also caused a significant increase in Ca^2+^ responses compared to control cells but TECC responses were clearly suppressed compared to cells pre-treated with 10 μg/mL. This suggests that apart from increasing Ca^2+^ influx, polygodial may also disrupt (affect) components of glucose signaling at high concentration. The finding that, at 50 μg/mL, polygodial TECC responses were completely inhibited, supports this hypothesis. In addition, at 50 μg/mL polygodial baseline, Ca^2+^ values were increased but the Ca^2+^ transient following membrane permeabilization was not affected compared to cells pre-treated with 10 or 25 μg/mL polygodial. 

## 3. Discussion

A chemogenomic screen examines a drug’s MOA by measuring the effect of the drug treatment on a collection of genetically distinct strains. According to Parsons et al., gene deletions that render strains hypersensitive to a drug facilitate the identification of pathways and proteins that buffer the cells against toxic effects of the drug, providing clues into its mode of action [50]. On the other hand, in a chemical–genetic interaction where the target gene of a compound is deleted, resistant phenotypes emerge as the target (gene product) to be affected by the compound. This is normally illustrated by screening with higher compound concentrations than the IC_50_ of the compound on the WT strain. Combining individual drug–mutant relationships (i.e., resistance or sensitivity) into a profile provides a genome-wide view of a compound’s effect on the cell [51]. 

To determine the antifungal MOA of polygodial, the model organism *S. cerevisiae* was used [28]. We obtained two sets of drug-dependent phenotypes: i.e., hypersensitive and resistant strains, using 2 and 6 µg/mL polygodial, respectively. In the hypersensitive group, vacuolar V-ATPase-related processes such as ATP hydrolysis-coupled proton transport, vacuolar acidification, and proton homeostasis were highly enriched (Table 1). These processes are critical for intracellular cytosolic and vacuolar pH regulation, and consequently, organellar acidification, in both *S. cerevisiae* and *C. albicans* [52,53]. Organellar acidification has been implicated in stress response, protein sorting in the biosynthetic and endocytic pathways, chitin localization, proteolytic activation of zymogen precursors, degradative (autophagic) processes carried out by the vacuole, storage of certain small molecules like Ca^2+^, and osmoregulation, in both *S. cerevisiae* and *C. albicans* [54,55,56]. In accordance with these stated functions, we also observed enrichment of hypersensitive mutants whose missing genes are responsible for protein sorting and recycling (Figure 2B and Table S1). Kondo et al. [25] concluded from their biochemical and growth inhibition study that the vacuolar membrane (on which the V-ATPase resides) is the primary target of polygodial. While it is clear that the vacuole is affected by polygodial treatment, our genomic screen shows that, in the absence of V-ATPase genes, the cells have a poor outcome when exposed to polygodial, rather than showing resistance as expected if the V-ATPase were the direct target. Thus, the vacuole regulates and buffers the cells against toxic effects of polygodial. From our results, it is highly likely that the distorted vacuoles seen by Kondo and colleagues are a secondary effect of polygodial. 

Polygodial treatment clearly affected yeast Ca^2+^ homeostasis (Figure 4). At 10 μg/mL, polygodial increased Ca^2+^ transients associated with addition of external Ca^2+^, re-addition of glucose (TECC response), and membrane permeabilization while in Ca^2+^-free medium supplemented with ethylene glycol tetraacetic acid (EGTA), cytosolic Ca^2+^ returned to baseline levels. Together, these results indicate that polygodial induces a rapid and sustained Ca^2+^ influx across the plasma membrane, leading to enhanced organellar Ca^2+^ storage. These findings are, therefore, compatible with the idea that polygodial mainly acts as a surfactant, disrupting plasma membrane lipid-protein interactions. At 25 μg/mL polygodial Ca^2+^ transients associated with external Ca^2+^ addition or membrane permeabilization remained elevated, however, TECC responses displayed lower peak and slower recovery. Finally, at 50 μg/mL, TECC responses were fully suppressed and basal Ca^2+^ levels increased. These results therefore suggest that at concentrations >10 μg/mL, polygodial may not only modulate Ca^2+^ homeostasis but also target glucose and TORC1 signaling. 

From the resistant mutants, we could define several processes that contribute to the resistant phenotype. These include cytoskeleton reorganization and endocytosis, the assembly of ribosome subunits and translation, respiratory gene expression mediated by the HAP complex, protein ubiquitination, and TORC1 signaling. The actin cytoskeleton not only acts as a structural scaffolding in the cell, thereby determining the size, shape, and mechanical properties of the cell, but also functions in endocytosis, exocytosis, polarized cell growth, cytokinesis, cell motility, and translation [57]. There are several observations that link the actin cytoskeleton to the V-ATPase. For instance, mutations in the gene encoding the stator V-ATPase E subunit (Vma4p) in *S. cerevisiae* have been shown to cause changes in cell morphology and cytoskeletal actin, probably mediated by intracellular pH or Ca^2+^ changes [58]. Moreover, Zhang and coworkers noticed that at high pH, abnormal bud morphologies and delocalization of actin and chitin occur [59]. Taken together, these data indicate that disruption of the cytoskeletal architecture due to polygodial can have a direct negative effect on the V-ATPase, the vacuole, and other cellular functions. 

The HAP complex consists of 4 proteins: Hap2p, Hap3p, Hap4p, and Hap5p, which positively control the expression of genes involved in mitochondrial respiration, mitochondrial function and biogenesis, and mitochondrial translation. Specifically, the HAP complex controls the complete TCA cycle, the electron transport chain, and related pathways [60]. Both the group of Castelli [22] and Lunde and Kubo [21] proposed mitochondria i.e., the electron transport chain and mitochondrial ATPase, respectively, as a target of polygodial. Buschlen et al. [60] indicated that the aforementioned components are part of the 5 respiratory chain complexes of *S. cerevisiae*. It is thus likely that the HAP complex plays a key role in polygodial resistance by controlling cellular respiration. Moreover, the HAP complex senses cellular redox status [61], which is affected by polygodial [24]. 

Most importantly, our analysis of the resistant mutants identified ubiquitin and TORC1 signaling as central nodes linking several of the aforementioned processes. Indeed, apart from the regulatory links already mentioned above with endocytosis, ribosome biogenesis, the HAP complex, and Ca^2+^ signaling, both ubiquitin and TORC1 are known to impact vacuole functioning and vacuolar biogenesis [62]. Moreover, TORC1 inhibition is associated to enhanced V-ATPase assembly [63] and the upregulation of autophagy [64], both processes that when failing lead to polygodial sensitivity. In addition, downregulation of TORC1 is associated with an enhanced oxidative phosphorylation (OXPHOS) density and respiration uncoupling [65,66,67], a mechanism that was previously proposed to underlie the effect of polygodial [22]. Hence, we propose that TORC1 and its protective agent ubiquitin may be central targets in the MOA of polygodial. Whether this is a direct effect or mediated by the second messenger Ca^2+^ remains to be clarified. 

In conclusion, we have shown that the vacuolar ATPase is involved in the antifungal action of polygodial in the model system *S. cerevisiae* by buffering the cell against a polygodial insult. The initial evidence from the vacuolar pH experiments and Ca^2+^ measurements implicate polygodial further as affecting this organelle. The cytoskeletal proteins have emerged as an interesting link to the vacuolar V-ATPases. Their role as a possible target of polygodial remains to be confirmed. We propose that the target for polygodial may act on TORC1 signaling, either directly or via disturbance of the Ca^2+^ homeostasis. In essence, our genetic screen has further increased the understanding of polygodial’s MOA and has shed light on underlying mechanisms and connections to previously reported observations. Finally, although we used *S. cerevisiae* to infer polygodial’s MOA in *C. albicans* since the two share about two-thirds of their open reading frames [29], further studies in *C. albicans* (e.g., using the CRISPR-Cas9 system) are needed to confirm these observations in pathogenic fungi.

## 4. Materials and Methods

### 4.1. Chemical Genetic Screen

The *S. cerevisiae* haploid *MAT*α (BY4742) deletion library, consisting of 4828 deletion mutants each lacking one specific gene, was used to determine genes that may be involved in the MOA of polygodial. Using a pinner, the strains stored in 20% glycerol and YP broth in 96-well plates at −80 °C were transferred to YPD-containing microtiter plates and incubated at 30 °C for 2 days. The strains were then diluted (1:20) and spotted on agar medium containing polygodial and incubated at 30 °C for 2 days. Throughout the screening process, we used minimal synthetic medium (SD) containing 0.5% ammonium sulfate and 0.17% Difco yeast nitrogen base without amino acids, supplemented with 2% glucose and histidine, lysine, leucine, and uracil (henceforth called SD_HLLU_). The amino acid concentrations used are as recommended by Sherman [68]. The pH was adjusted to 5.5 when SD_HLLU_ broth was used. In the SD_HLLU_ agar medium (pH 6.5), two concentrations of polygodial, which had previously been established via pilot experiments (i.e., 2 and 6 μg/mL), were used to screen for hypersensitive and resistant strains, respectively, using growth as read-out. The WT strain (*S. cerevisiae* BY4742) was also spotted on each plate as a control. An empirical qualitative score from 0 to 6 was used to grade growth (reduced colony formation) in comparison to the WT strain: 0 = no growth, 1 = limited growth, 2 = moderate growth, 3 = similar growth as WT, 4 = slightly more growth than WT, 5 = denser growth than WT, and 6 = excellent growth compared to WT.

We used SGD GO Term Finder and FunSpec for the evaluation of groups of genes and proteins (e.g., co-regulated genes, protein complexes, genetic interactors) as defined by their annotation: e.g., functional roles, biochemical properties, or localization. FunSpec uses different databases, other online knowledge bases, and published datasets such as the Gene Ontology and Munich Information Center for Protein Sequences (MIPS) database. These are comprehensive genome databases that list functional classification, known protein complexes, protein classes, mutant phenotypes, and subcellular localization. The Bonferroni-correction used during analysis divides the p-value threshold that would be deemed significant for an individual test, by the number of tests conducted, and thus accounts for spurious significance due to multiple testing over the categories of a database. Additionally, the STRING database was used to visualize protein–protein interactions. 

### 4.2. Calcium Measurements Using an Apoaequorin System

The measurements were based on the method described by D’hooge et al. [49]. Briefly, *S. cerevisiae* BY4741 (*MAT*a *his3Δ1 leu2Δ0 met15Δ0 ura3Δ0*) cells were transformed with the plasmid (pYX212-cytAEQ) containing the apoaequorin gene, and transformants were selected for growth on SD medium with 2% glucose, lacking leucine. Cells were then grown overnight at 30 °C and harvested when the OD_600_ reached 2. The yeast cells were then immobilized for 1 h on concanavalin A-coated coverslips in 12-well culture plates in fresh SD medium with 2% glucose, lacking leucine. Later, when the medium was removed, the cells were washed with 0.1 M 2-(N-morpholino) ethanesulphonic acid (MES) and incubated again for 1 h at 30 °C in 0.1 M MES/ trisaminomethane (TRIS) pH 6.5 supplemented with 5 μM coelenterazine (Promega) to charge aequorin. Polygodial was added at final concentrations of 10, 25, and 50 µg/mL for 30 min. Excess coelenterazine was removed by washing the cells. The coverslips were mounted in a thermostated superfusion chamber (30 °C). The glucose-starved yeast cells were initially superfused with 0.1 M MES/TRIS (0–1 min), pH 6.5, followed by 0.1 M MES/TRIS pH 6.5 supplemented with 10 mM CaCl_2_ (referred to as Ca^2+^ pulse) from 1–3 min. Cells were then stimulated by the addition of 80 mM glucose (3–7 min) to induce a transient elevation of cytosolic Ca^2+^ (TECC) response. To estimate intracellular Ca^2+^ storage from organelles, cells were exposed to a Ca^2+^-free medium containing 3 mM EGTA (7–8.5 min), and permeabilized with 0.5% Triton X-100 in the same medium (8.5–12 min). At the end of each experiment, cells were superfused with a Ca^2+^-rich hypotonic medium (10 mM CaCl_2_ in H_2_O). Photons emitted as a result of Ca^2+^ binding to charged aequorin were continuously detected by a photon-counting tube (Type H3460-04, Hamamatsu Photonics, Japan) that was positioned about 2 cm above the cells. Light impulses were pre-scaled and counted with a PC-based 32-bit counter/timer board (PCI-6601, National Instruments Corporation, Austin, TX, USA). The number of impulses occurring during a 1 sec time interval was monitored with custom-built software. The recorded aequorin luminescence data were calibrated offline into cytosolic Ca^2+^ ([Ca^2+^]_in_) values using the following equation: [Ca^2+^]_in_ = ((*L*/*L*_max_)^1/3^ + [118(*L*/*L*_max_)^1/3^ − 1)/(7 × 10^6^ − [7 × 10^6^(*L*/*L*_max_)^1/3)^]), where *L* is the luminescence intensity at any time point and *L*_max_ is the integrated luminescence. The experiment was repeated three times.

### 4.3. Vacuolar pH Measurements

The measurements were performed according to Diakov et al., with some modifications [69]. Briefly, WT *S. cerevisiae* BY4742 was grown overnight in 50 mL SD_HLLU_ containing 50 mM MES to OD 2.0. The cells were then transferred to a 50 mL Falcon tube and centrifuged at 2061 g for 5 min. The supernatant was discarded and the cells were resuspended in 250 µL LoFlo SD_HLLU_ medium containing 50 µM 2’7’-Bis-(2-carboxyethyl)-5-(and 6)-carboxyfluorescein (BCECF-AM), then incubated at 30 °C for 30 min. While the cells were incubating, calibration buffers were prepared. These buffers contain cell-permeant ammonium acetate capable of collapsing pH gradients across multiple membranes, sodium azide and deoxyglucose to halt ATP production and inhibit the H^+^-pumps. The cells were then washed twice with the LoFlo medium and 20 µL of the cells was transferred to each well of a multiwell plate containing 180 µL medium or calibration buffer. The plates (Greiner, black Ref# 655209) were then measured in a Fluoroskan apparatus with filters for BCECF-AM, alternately measuring at λ_ex_ 440 and 485 nm, and λ_em_ at 538 nm. Baseline measurements were first taken for 1 h in the presence of calibration buffers covering a pH range of 4.5–7, then polygodial (final concentration 5, 15, and 45 µg/mL) and DMSO were added in their respective wells, and the plate was further measured for 30 min. To obtain a calibration curve, the ratio of fluorescence at 485 to 440 nm for each calibration buffer was calculated for each tested condition, then using the fluorescence ratios obtained with treated cells, vacuolar pH was calculated using the respective standard curves. The vacuolar pH was then plotted against time. The experiment was repeated twice. 

## Figures and Tables

**Table 1:** Analysis of gene enrichment in the pools of polygodial sensitive and resistant strains based on the FunSpec software. Genes shown in bold correspond to hypersensitive or hyper-resistant strains. K denotes the number of genes from the input (sensitive or resistant strains) in a given GO category, while F refers to the total number of genes encoded by the yeast genome in a given GO category.

Polygodial Sensitive			
Process	Genes in Cluster	K	F
ATP hydrolysis coupled proton transport	***VMA1 VMA3 VMA8 VMA7 VMA16 ATP2 VMA4 VMA13***	8	17
vacuolar acidification	***VMA1 VMA3 VMA8 VMA7 VMA16 VPH2 VMA4 VMA13 ***	8	26
piecemeal microautophagy of nucleus	***SHP1** ATG8 ATG12 **VAM6 VAM7** ATG10 **VAM3** ATG13*	8	33
cellular amino acid biosynthetic process	***TYR1 TRP1 PRO1 TRP2 TRP5 ADE3 SER2 ARG2 SER1 CPA1 PRO2***	11	98
nucleosome mobilization	*SNF6 SWI3 **ARP5 SNF12 TAF14***	5	16
transcription mediator complex	***ROX3** MED2 SRB5 **SRB2** CSE2 GAL11 SSN3 **TAF14***	8	26
regulation of transcription, DNA-dependent	***ROX3 HTL1** TUP1 MED2 **RPN4** SAC3 UME6 EAF1 **ADA2** RTF1 SRB5 SMI1 SNF6 **SRB2 SWI3GON7 BUR2** CHS5 ARP5 CSE2 **SNF12** GAL11 ULS1 **SSN3 TAF14***	25	507
retrograde transport, endosome to Golgi	*RGP1 VPS35 **VPS52 VPS54 YPT6***	5	18
** Polygodial Resistant**			
**Process**	**Genes in Cluster**	**K**	**F**
CCAAT-binding factor complex	***HAP2 HAP4 HAP5***	3	4
GSE/EGO complex	***GTR1 GTR2** LTV1*	3	5
cytoskeletal protein binding	***SLA1 RVS161 RVS167 SRV2***	4	7
structural constituent of ribosome	***RPL1B** RPS1B **RPL13A** RPS16A **RPS16B** RPL16A RPS17B RPL19A RPL41B RPL27B RPL23A RPL23B RPL24A RPL24B RPS25A RPS28B RPL29 RPS30A **RPL37A** RPP2A **MRPL35** MRPL38 **RSM27***	23	218
mitochondrial matrix	*MSK1 MTF2 **MSE1** TUF1 CPR3 MDJ1 **FUM1** PDX1 CIT1 **ALT1 AAT1** ACO1 SDH5*	13	111

## Data Availability

All data is presented in the Supplementary section.

## Supplementary Materials

The following are available online at https://www.mdpi.com/article/10.3390/ijms22115756/s1.
