# Supplementary material for: Investigating the Antifungal Mechanism of Action of Polygodial by Phenotypic Screening in Saccharomyces cerevisiae
Source: Int J Mol Sci. 2021 May 28;22(11):5756. doi: 10.3390/ijms22115756 (PMC8198865; doi:10.3390/ijms22115756)

International Journal of *Molecular Sciences*

MDPI

**Supplemental Tables and Figures**

**Table S1**: Deletion strains sensitive (n=135) to polygodial ranked as 0 and 1. In bold are hypersensitive mutants (n=66). Gene functions and cellular locations were taken from the SGD database.

| Biological process | Genes |
|---|---|
| Mitochondrial genome maintenance and normal morphology | *GET1*, *PCP1*, ***YME2*** |
| Mitochondria electron transport chain | ***QCR10*** |
| Mitochondria ATPase | ***ATP2*** |
| Mitochondrial protein maturation | ***ISA1*** |
| Mitochondrial import,folding, maturation, degradation | *YME1* |
| Mitochondrial TCA cycle | *KGD2* |
| Mitochondrial NADP biosynthesis | *POS5* |
| Mitochondrial riboflavin biosynthesis | ***RIB4*** |
| Peroxisome protein deubiquitination | ***UBP15*** |
| Vacuolar acidification, V-ATPase | ***VMA1***, ***VMA3***, ***VMA4***, ***VMA7***, ***VMA8***, ***VMA13***, ***VMA16***, ***VPH2*** |
| Vacuolar degradation autophagy | *ATG8*, *ATG12*, *ATG10*, *ATG13* |
| Vesicular trafficking to vacuole, vacuolar protein sorting | ***VAM6***, ***VAM3***, ***GYP1***, ***VAM7***, ***VPS16***, ***VPS54***, *VPS61*, *VPS63*, *VPS65* |
| Golgi P-type ATPase | ***PMR1*** |
| ER Golgi endosome trafficking | *CHS5*, *RGP1*, ***YPT6***, *VPS35*, ***VPS52***, ***VPS54,*** *SEC22*, *SEC28*, *YSP1* |
| ER protein targeting to membrane, translocation | *SEC66*, *SGT2*, *GET1*, ***GET4*** |
| ER Golgi protein N-linked glycosylation | ***OST4***, *OCH1* |
| ER ergosterol biosynthesis | ***ERG3***, *ERG2*, *ERG28* |
| ER sterol transport | ***SIP3*** |
| ER lipid droplet formation and triacelglyceride homeostasis | *VPS66/LOA1* |
| ER glycosylphosphatidylinositol biosynthesis | ***LAS21*** |
| ER-associated degradation (ERAD) | ***SHP1*** |
| Nuclear enveloppe phospholipid biosynthesis, lipid droplets | *NEM1* |
| Chromatin organization, structure, remodelling | *EAF1*, *ULS1*, *RTF1*, ***ARP5*** |
| Plasma membrane transport | ***FUR4,*** *TRK1* |
| Cell wall synthesis | *SMI1*, *FKS1* |
| Bud site selection & axial budding | ***BUD25*** |
| Cytoskeleton organization and cellular morphogenesis | *BEM2* |
| Actin stabilization | ***TPM1*** |
| Transcription regulation | *SWI3*, ***TAF14***, ***SSN3***, *GAL11*, *CSE2*, ***SIP3***, ***BUR2***, ***GON7***, ***ROX3***, ***SRB2***, *SNF6*, ***SNF12***, *SRB5*, *RTF1*, ***ADA2***, *UME6*, ***RPN4***, *MED2*, *TUP1*, *RPA14* |

| | |
|---|---|
| Mitosis, meiosis | *DCC1*, *CTF4*, *CNM67*, *VPS54*, ***HTL1***, ***CIK1***, *MRE11* |
| mRNA export from nucleus | *SAC3*, ***NPL3*** |
| Nuclear protein import | ***MOG1*** |
| DNA repair | *FYV6* |
| Amino acid synthesis | ***TYR1***, ***CPA1***, ***ADE3***, ***PRO1***, ***PRO2***, ***SER1***, ***SER2***, ***TRP1***, ***TRP2***, ***TRP5***, ***CPR2***, ***ARG2*** |
| Ribosomal protein; translation and protein synthesis | *RPL13B*, ***MRPL23***, ***TIF3***, *CPA1 uORF* |
| Ribosome biogenesis | *SAC3*, *BMT5* |
| Signal transduction | *TPK1*, ***IRA2***, ***PHO85***, *CLA4*, *NBP2*, *STE11* |
| Glycolysis | ***PFK2***, *ADH1* |
| Glutathione biosynthesis, binding | ***GSH1***, *URE2* |
| Cyanamide metabolism | ***DDI3*** |
| Trehalose synthesis | *TPS2* |
| Nucleotide metabolism | *ADE3*, *ADK1* |
| Intracellular copper transport | *CCS1* |
| Mating | *SCP160* |
| Unknown function | *ECM33*, *BOP2*, *JJJ2*, *YGL024W*, *YML012C-A*, *YER091C-A*, *YDR433W*, *YDR157W*, *YMR102C*, *YOR331C*, *YNL296W* |

**Table S2**: Mutants resistant to polygodial ranked as 5 and 6 (n=233). In bold are hyper-resistant mutants (n=47). Gene functions and cellular locations were taken from the SGD database.

| Biological process | Genes |
|---|---|
| Mitochondrial transcription and translation | *PET494*, *MSK1*, *MEF1*, *MEF2*, *MTF2*, *MRM2*, *MRPL38*, ***MRPL35***, ***SLS1***, *TUF1*, ***MSE1***, ***RSM27***, *MRX5* |
| Mitochondrial genome maintenance, fusion, fission and normal morphology | ***MCP2***, *CCE1*, *RIM1*, *ACO1*, ***MGM1***, ***DNM1***, *UGO1* |
| Mitochondria ATPase | *ATP15*, ***ATP17***, ***AFG3*** |
| Mitochondrial inner membrane electron transport chain | ***QCR9*** |
| Mitochondrial protein folding | *CPR3*, *MDJ1* |
| Mitochondrial TCA cycle | ***FUM1***, *PDX1*, *CIT1*, *SDH5*, *ACO1*, *ACO2* |
| Mitochondrial amino acid biosynthesis | ***GDH3***, ***ALT1***, *THI4*, *AAT1* |
| Mitochondrial transporter activity | *MDL1*, *HOT13*, *FLX1*, *MRS2* |
| Mitochondrial lipid homeostasis | *UPS2*, *MCP2* |
| Mitochondrial unknown function | *MRP8*, *FMP33*, *FMP48*, *FMP49* |
| Peroxisome biogenesis | *PEX30* |
| Vacuolar protein sorting, MVB, ESCRT, CORVET | *SNA3*, *MIL1*, *CPS1*, *YCK3*, ***VPS8***, ***VPS9***, *VPS21*, *VPS36*, *VPS60*, *VPS25*, *VPS27*, *MVB12*, *SSH4*, *LST4*, *ENV7* |
| Vacuolar transport | *AVT3*, ***BPT1***, *CCC1* |
| Vacuolar carboxypeptidases | *ATG42*, *CPS1* |
| Golgi glycosylation | ***MNN4*** |
| ER Golgi transport | *COY1* |

| | |
|---|---|
| ER enzymes and transport | *ICE2, ERV2, YBR137W* |
| ER cargo exit | *TED1* |
| ER lipid homeostasis | ***OSH2***, *APQ12* |
| ER inheritance | *SHE3* |
| Actin cytoskeleton reorganization, microtubules and endocytosis | ***RVS167***, ***RVS161***, ***SLA1***, ***END3***, ***ACF2***, ***SRV2***, *GVP36, TPM2, PAC11, CIN1* |
| Plasma membrane transport | *QDR1, AQR1, HXT10, ALR2, FRE7, BOR1, AMF1,* |
| Cell wall | *FIT3, KDX1, PRY3, TOS1* |
| Bud site selection & axial budding | *BUD2*, ***BUD20***, *BUD21, BUD26, BUD28, AXL2* |
| Mating | *FUS3, KDX1, PRY3* |
| Mitosis and Meiosis | *HOP1, CLG1, IRC8, RMR1, HOS4, REC8, SMA2, GMC1* |
| Nuclear protein, protein quality control | *CMR1* |
| DNA repair and excision | *RAD7, RAD16, EAF7, MMS4, REV7, UBC13, YNK1* |
| Transcription regulators | *MSS11, TEA1, RFX1, MRK1*, ***HAP4***, *CSI1, URC2, SPT7, HAA1*, ***EAF7***, ***HAP2***, *NOT3*, ***HAP5***, *YAP7, TYE7, ACM1, HOS2, LAG2* |
| RNA processing | *NNF2, MUD1, CAF40, PES4* |
| Ribosomal protein; translation and protein synthesis | *RPP2A, RPL24B*, ***RPL37A***, ***RPS28B***, *RPL29, RPL24A, RPL23A, RPL23B, RPL16A, RPL41B*, ***RPL13A***, ***RPS16B***, *RPS17B, RPS16A, RPL27B, RPL19A, RPS25A*, ***RPL1B***, *RPS30A, RPS1B* |
| Ribosome biogenesis | *SYO1, NOP12, GVP36* |
| Signal transduction, GSE/EGO complex | ***MDS3, GTR1, GTR2,*** *LTV1, PIB2*, ***CKA2,*** *RCN1, CNB1*, ***SIP5,*** *GIS4* |
| Ubiquitination & proteasome complex activity | ***UBI4***, ***UBR2***, ***UBC13***, *PRE9, MMS2*, ***MUB1***, ***NPL4***, *UBR1, HUL5, ELA1* |
| Nucleotide biosynthesis | *YNK1, URA4, AMD1* |
| Cytoplasmic L-methionine salvage | *UTR4* |
| Glycogen biosynthesis, glycolysis and gluconeogenesis | ***TDH3***, *GSY2* |
| Glycerol metabolism | *GPD2* |
| NADH-dependent aldehyde reductase | *YNL134C* |
| NAD biosynthesis | *YEF1* |
| Undefined/ unclear/unknown | ***IRC19***, *OCA4*, ***OCA5***, *GDS1, FHN1, STB6, NQM1, PBP4, FMP33, FMP48, BSC4, CMR3, CMG1, SDD2*, YPR078C, YOR333C, YOL079W, YOR309C, YNR005C, YML084W, YMR141C, YML083C, YKL147W, YLR407W, YLR169W, YLR125W, YLR184W, YLL047W, YKL075C, YJR056C, YIL161W, YJL028W, YIL141W, YGR219W, YGR137W, YGR122C-A, YFL032W, YFL015C, YER038W-A, YDR442W, YBR013C, YDL094C, YDL062W, YBR287W, YML095C-A, YBR144C, YBR138C, YJL017W, YJL206C-A, YBR096W, YGR153W |

**Figure S1**: An example of a read-out from the chemogenomic screen. The same mutant strains from a 96-well plate were spotted on these three agar plates. The top agar plate contains DMSO (diluent of polygodial) and is a control plate. The middle agar plate contains 2 μg/mL polygodial (to detect hypersensitive strains) while the bottom plate contains 6 μg/mL polygodial (to detect resistant strains). Ranking was based on the growth of the mutants relative to the WT BY4742. Lower ranks (0-2) represent poor growth, 3 represents WT and more growth than WT ranks from 4-6.

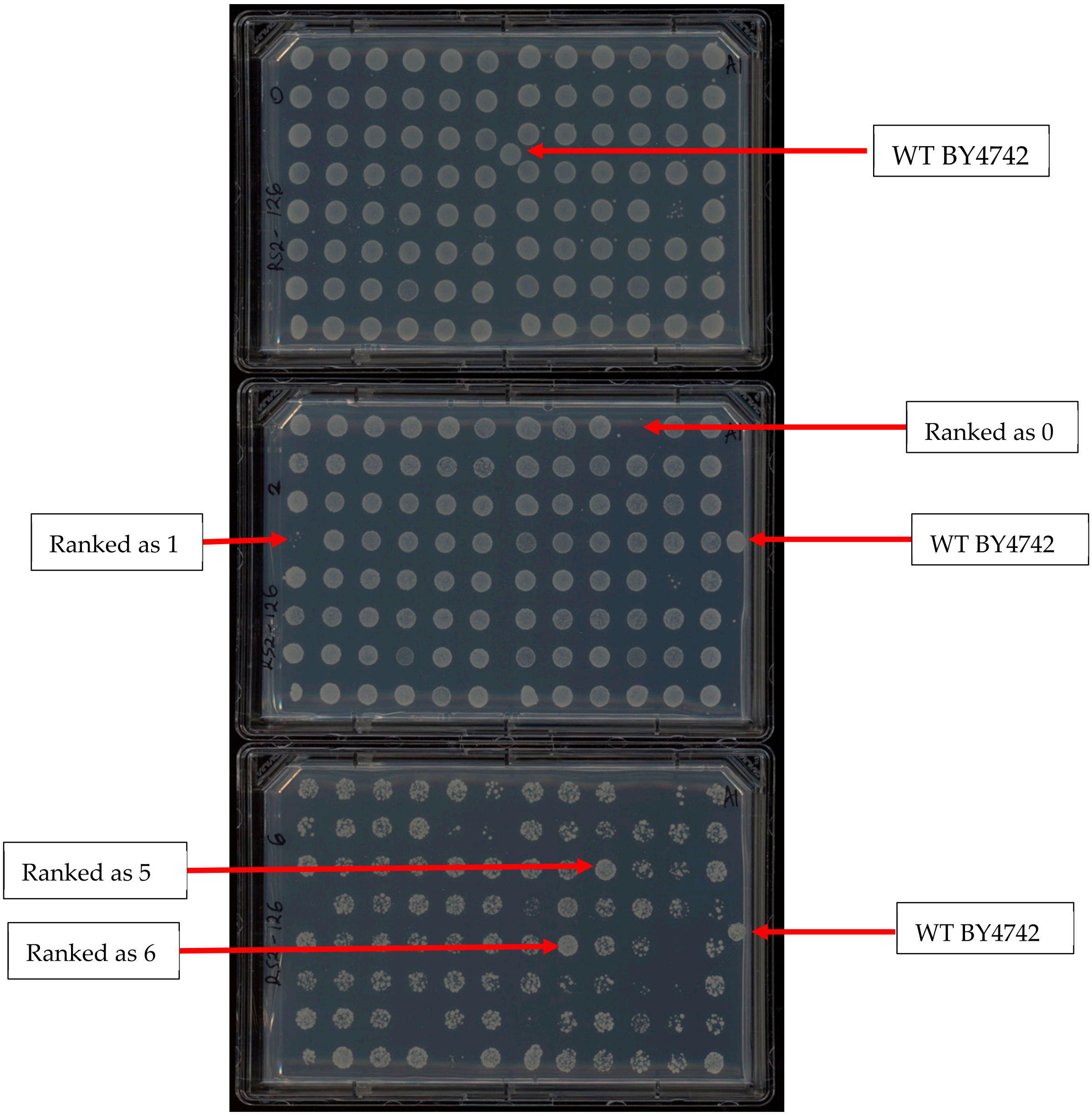

**Figure S2**: A clustering visualization of all polygodial sensitive strains using the STRING database. The colored circular nodes represent proteins, while the edges joining the nodes represent interactions (protein-protein), either established known interactions or predicted interactions.

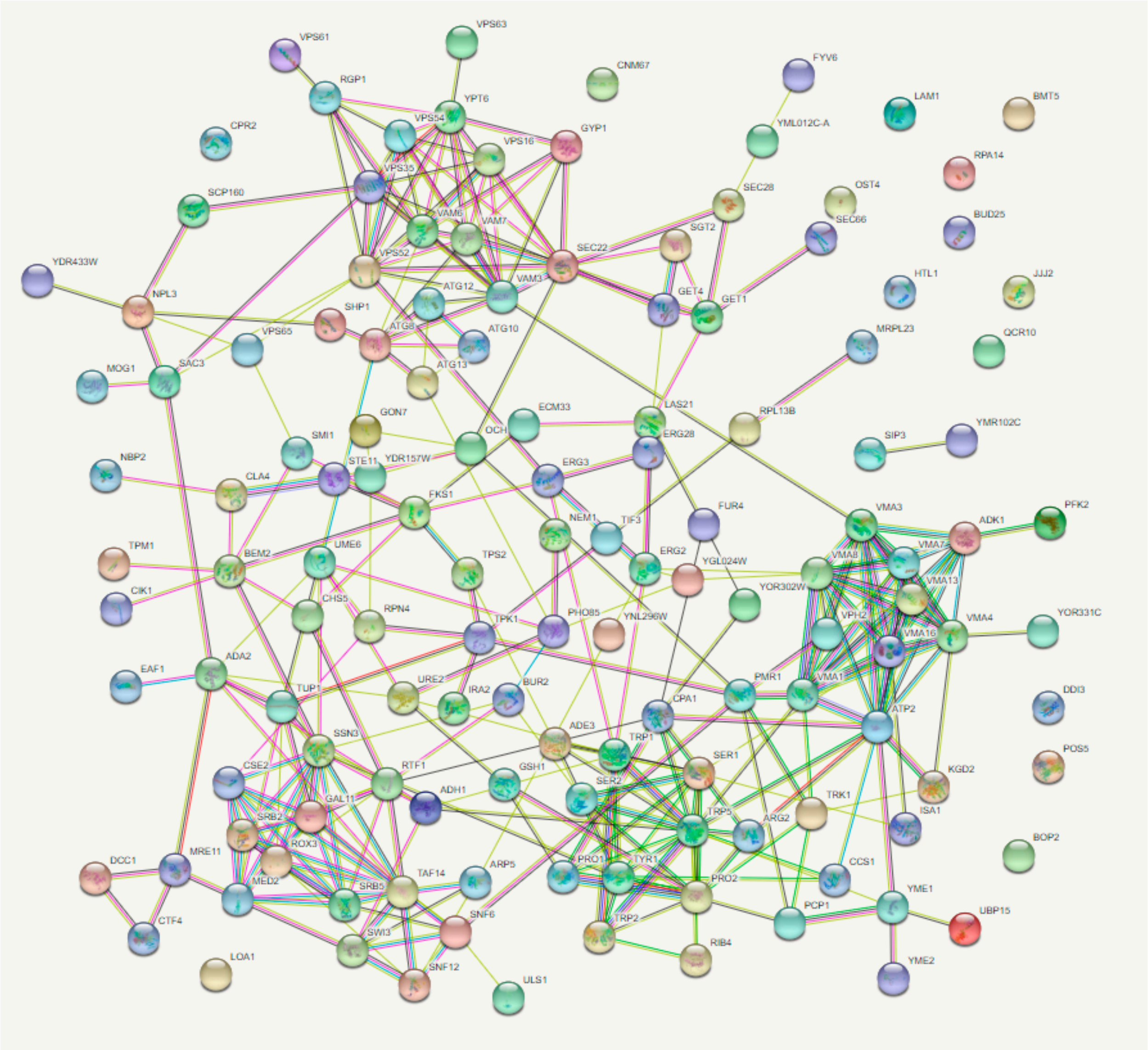

**Figure S3**: A clustering visualization of all polygodial resistant strains using the STRING database. The colored circular nodes represent proteins, while the edges joining the nodes represent interactions (protein-protein), either established known interactions or predicted interactions.

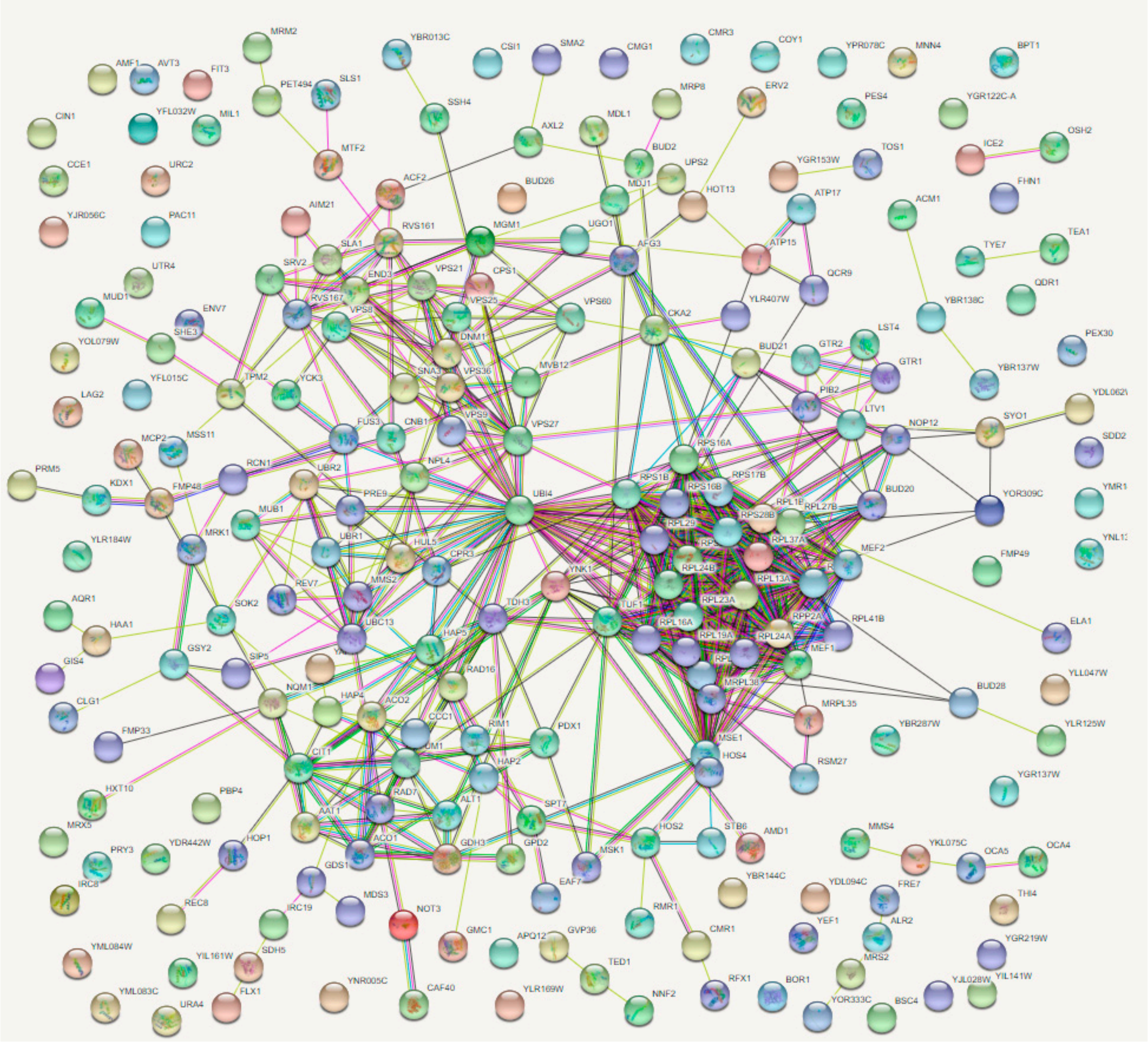

Supplement: Supplementary file 1 [file ijms-22-05756-s001.zip › Supplementary File ijms-1210848.pdf]
